# The significance of aspartate on NAD(H) biosynthesis and ABE fermentation in *Clostridium acetobutylicum* ATCC 824

**DOI:** 10.1186/s13568-019-0874-6

**Published:** 2019-09-10

**Authors:** Zhengping Liao, Xitong Yang, Hongxin Fu, Jufang Wang

**Affiliations:** 1 School of Biology and Biological Engineering, South China University of Technology, Guangzhou, 510006 China; 2 State Key Laboratory of Pulp and Paper Engineering, South China University of Technology, Guangzhou, 510640 China

**Keywords:** *Clostridium acetobutylicum*, Butanol, Aspartate, NADH

## Abstract

The co-factor NADH plays an important role in butanol biosynthesis. In this study, we found that aspartate could effectively improve the butanol production of *Clostridium acetobutylicum* ATCC 824. Further study showed that aspartate could be used as the precursor of NADH de novo synthesis in *C. acetobutylicum* ATCC 824. When 2 g/L aspartate was added, the transcription levels of essential genes (*nadA*, *nadB* and *nadC*) for NADH de novo synthesis were significantly higher than that of without aspartate addition. The levels of intracellular NAD^+^, NADH, total NAD(H) and the ratio of NADH/NAD^+^ were also significantly increased, which were 63.9 ± 8.0%, 85.0 ± %, 77.7 ± 8.0% and 12.7 ± 2.9% higher than those of without aspartate addition, respectively. Furthermore, the butanol production was improved by overexpressing the NADH de novo synthesis genes, and the fermentation performance could be further enhanced by strengthening the VB1 biosynthesis and NADH de novo synthesis pathway simultaneously. As a result, the butanol titer of the engineered strain 824(thiCGE–nadC) reached 13.96 ± 0.11 g/L, 7.2 ± 0.4%, 18.1 ± 0.1%, 34.1 ± 0.1% higher than that of 824(thiCGE), 824(nadC) and the wild type strain, respectively. This study has a reference value for the NADH related researches of other microbes, and the engineering strategy used in this study provides a new idea for construction of efficient fuel-producing strains.

## Introduction

With the exhaustion of fossil energy and the deterioration of global environmental problems, the biofuels have been attracted more and more attention. As a biofuel, the physical and chemical properties of butanol are superior to ethanol and similar to gasoline, thus it could be used as a fuel additive or as a replacement of gasoline (Lee et al. 2008). However, the market competitiveness of biobutanol is weak due to the low titer, yield and proportion of butanol in traditional ABE fermentation. Therefore, improving the butanol production is of great significance to the butanol fermentation industry.

In organisms, most of the physiological and biochemical processes are achieved through redox reactions. Cofactor NADH is essential in these redox reactions. For example, NADH was involved in 43 reactions in *Escherichia coli* and 65 reactions in *Saccharomyces cerevisiae* (Nielsen 2003). Therefore, redox balance is of great significance for maintaining normal cell growth and physiological metabolism, and NADH plays an important role in maintaining the redox balance (Qi et al. 2018). In addition, NADH also could affect the products formation and metabolic flux redistribution. For example, the succinic acid production by *E. coli* and the ethanol production by *Clostridium thermophilus* can be improved by increasing the supply of NADH (Balzer et al. 2013; Biswas et al. 2015). It was reported that NADH is one of the key factors in the transformation from acidogenic phase to solventogenic phase of ABE (acetone–butanol–ethanol) fermentation by *C. acetobutylicum*, and the redox balance could determine the metabolic flux redistribution (Peguin et al. 1994; Wietzke and Bahl 2012). In *C. acetobutylicum*, the biosynthesis of butanol is strongly dependent on the availability of cofactor NADH. In general, the synthesis of butanol is an NADH consuming process: 1 mol of glucose can produce 2 mol of NADH through glycolysis, while 4 mol NADH are required for 1 mol of butanol synthesis. The NADH produced in glycolysis cannot meet the demand of butanol synthesis, thus insufficient NADH supply have limited the synthesis of butanol. Therefore, it is an effective way to improve butanol production by increasing the NADH supply in *C. acetobutylicum*. In recent years, a lot of work in improving the concentration of NADH for biofuels production have been reported. Formic acid dehydrogenase (FDH) can oxidize formic acid into carbon dioxide and generate NADH. Therefore, the NADH levels and butanol production were significantly improved by introducing FDH into *E. coli* (Lim et al. 2013; Shen et al. 2011). However, the NADH supply was not significantly affected after heterogenously expressed FDH from *Candida boidinii* in *C. acetobutylicum* (Wang et al. 2011). Hydrogenase has a great influence on NADH formation because it can compete with ferredoxin for electrons to produce hydrogen. Therefore, inhibiting the activity of hydrogenase could be used as an effective strategy to improve NADH supply. For example, Biswas’ research showed that knocking out the hydrogenase gene of *Clostridium thermocellum* resulted in an increasement of the ethanol titer and yield (Biswas et al. 2015). However, it is difficult to knockout hydrogenase gene in butanol producing *Clostridium*, or its effect on NADH formation and product distribution was very limited, even the hydrogenase gene knockout was achieved (Cooksley et al. 2012; Jang et al. 2014; Liu et al. 2015). In addition, the NADH and ATP levels of *Clostridium beijerinckii* NCIMB 8052 was significantly increased by insertional inactivation of the NADH-quinone oxidoreductase gene Cbei_4110, resulting in a 21.8% improved in butanol titer (Liu et al. 2016). NAD(P)H availability was also improved by overexpressing FdNR (ferredoxin NAD(P)^+^ oxidoreductase) in the *C. acetobutylicum* buk^−^ strain, which resulted in an improvement in the production of butanol and the ratio of butanol/acetate (Qi et al. 2018).

**Fig. 1:**
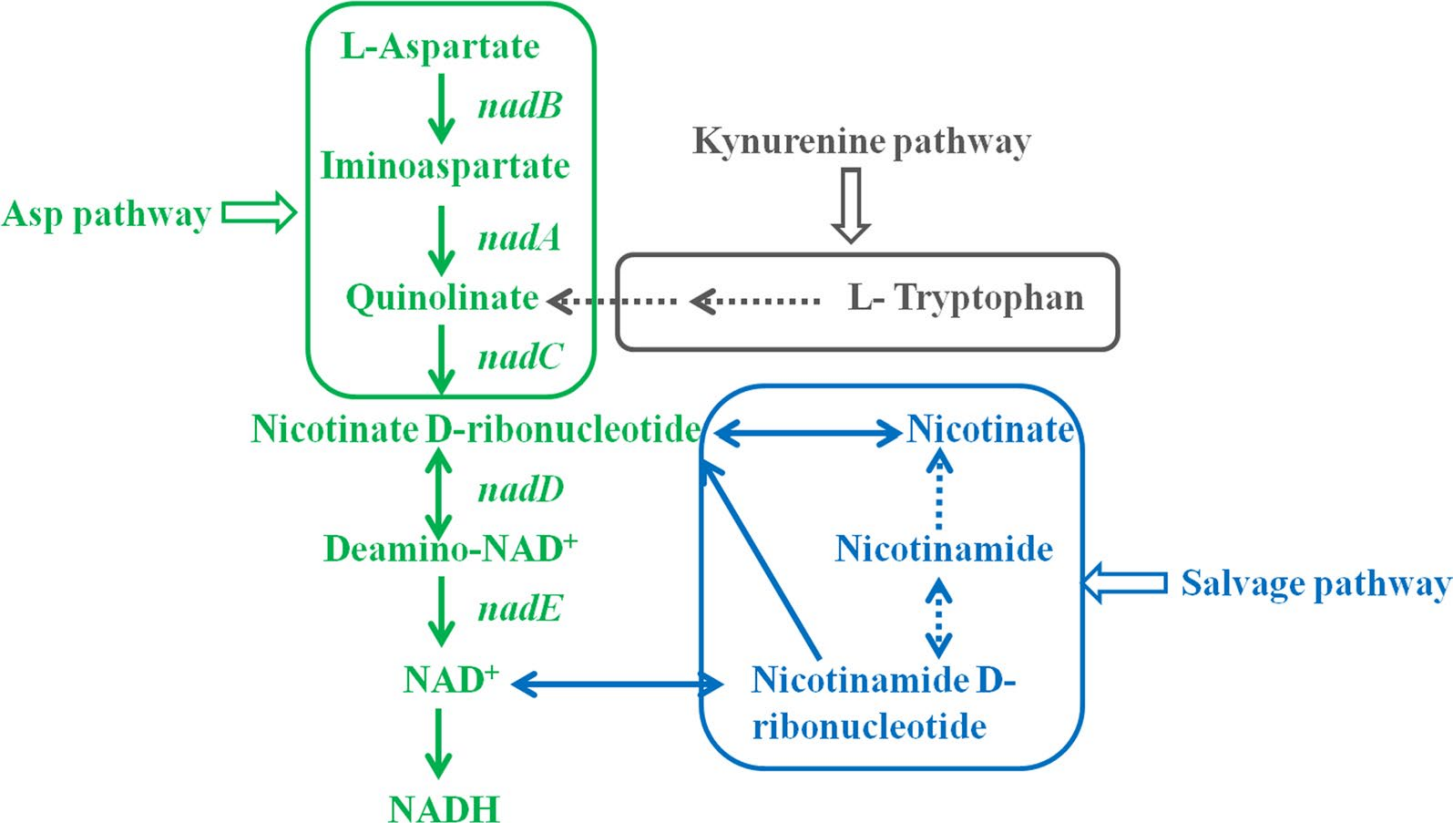
NADH biosynthetic pathway of *Clostridium acetobutylicum* ATCC 824. The gray section means that Kynurenine pathway does not exist in *Clostridium acetobutylicum* ATCC 824. *nadB*, l-aspartate oxidase gene; *nadA*, quinolinate synthetase gene; *nadC*, nicotinate-nucleotide pyrophosphorylase gene; *nadD*, nicotinic acid mononucleotide adenylyltransferase; *nadE*, NAD synthetase

However, there were few researches focused on increasing the NADH levels of solventogenic *clostridia* by enhancing the de novo synthesis pathways of NADH. In organism, NADH was synthesized by means of de novo pathway and salvage pathway (Fig. 1). There are two de novo synthesis pathways of NADH, namely Asp pathway and Kynurenine pathway. Asp pathway used aspartate as the precursor, and Kynurenine pathway used tryptophan as the precursor (Akira et al. 2006) (Fig. 1). The way of NADH de novo synthesis differs in different microorganisms. For example, in most eukaryotes (e.g., *Saccharomyces cerevisiae*, *Streptomyces antigens*), NADH is synthesized with tryptophan as the precursor (Kurnasov et al. 2003); while in most prokaryotes (such as *E. coli*), NADH is synthesized with aspartate as the precursor (Akira et al. 2006). Based on the genomic sequencing data (Nölling et al. 2001), there are no Kynurenine pathway genes in genome of *C. acetobutylicum* ATCC 824. Therefore, we speculated that the de novo synthesis of NADH in *C. acetobutylicum* ATCC 824 is by Asp pathway, rather than by Kynurenine pathway. As shown in Fig. 1, aspartate is catalyzed by aspartate oxidase (NadB), quinolinate synthetase (NadA) and nicotinate-nucleotide pyrophosphorylase (NadC) to form nicotinate d-ribonucleotide (NaMN). Subsequently, NaMN is catalyzed by nicotinic acid mononucleotide adenylyltransferase (NadD) and NAD synthase (NadE) to form NAD^+^, these two enzymes are common to both the de novo pathway and salvage pathway. Therefore, *nadA* (cac1025), *nadB* (cac1024) and *nadC* (cac1023) are essential genes for de novo synthesis of NADH in *C. acetobutylicum* ATCC 824.

In this study, the effect of aspartate on ABE fermentation and NAD(H) biosynthesis of *C. acetobutylicum* ATCC 824 was investigated firstly. Then, the essential genes (*nadA*, *nadB* or *nadC*) for NADH de novo synthesis were overexpressed to improve the butanol production. Moreover, to further increase the butanol production, both the biosynthesis pathway of NADH and VB1 (*thiC*, *thiG* and *thiE*) were enhanced in *C. acetobutylicum* ATCC 824. The results showed that both aspartate addition and NADH de novo synthesis genes overexpression could promote butanol production and NAD(H) biosynthesis. Moreover, co-expression of *nadC*, *thiC*, *thiG* and *thiE* could further improve the butanol production of *C. acetobutylicum* ATCC 824. This work revealed that enhancing Asp pathway could improve the butanol production of *C. acetobutylicum* ATCC 824 by promoting NAD(H) biosynthesis, providing a new and effective engineering target for solventogenic *Clostridia*.

## Materials and methods

### Strains, plasmids and primers

The strains and plasmids used in this study are shown in Table 1. The plasmid pMTL82151 is an *E. coli*–*Clostridium* shuttle plasmid, and the plasmid pAN2 contains a methyltransferase gene *Φ3t I*. *C. acetobutylicum* ATCC 824, which used as a wild type strain, was provided by Professor Shang-Tian Yang (The Ohio State University). All of *E. coli* strains were purchased from TIANGEN (Beijing, China). *E. coli* DH5α was used for plasmids amplification and *E. coli* TOP10 (containing pAN2) was used for recombinant plasmids methylation in vivo. All of the primers used in this study (Table 2) were synthesized by TIANYI HUIYUAN (Guangzhou, China).

**Table 1:** Strains and plasmids used in this study

Strains and plasmids	Characteristics	Sources and references
Strains		
*C. acetobutylicum* ATCC 824	Wild type	ATCC
*E. coli* DH5α	*DeoR, recA1, endA1, hsdR17* (rk^−^, mk^+^)	TIANGEN
*E. coli* Top10	*hsd R, mcr A, rec A1*, *end A1*	TIANGEN
Plasmids		
pAN2	Φ3t I, p15A ori, Tet^R^	Heap et al. (2007)
pMTL82151	ColE1 ori; Cm^R^; pBP1 ori; TarJ	Heap et al. (2009)
pMTL-Pthl	From pMTL82151; P-*thl*	Liao et al. (2018)
pMTL-Pthl thiCGE	From pMTL82151; P-*thl*::*thiC *+ *thiG *+ *thiE*	Liao et al. (2018)
pMTL-Pthl-nadA	From pMTL82151; P-*thl*::*nadA*	This work
pMTL-Pthl-nadB	From pMTL82151; P-*thl*::*nadB*	This work
pMTL-Pthl-nadC	From pMTL82151; P-*thl*::*nadC*	This work
pMTL-Pthl-thiCGE–nadC	From pMTL82151; P-*thl*::*thiC *+ *thiG *+ *thiE *+ *nadC*	This work

**Table 2:** Primers used in this study

Primers	Sequence (5′–3′)	Sources and references
Primers for gene amplification		
*nadA*-F	AGGAGGTTAGTTAGA GGATCC TCACATACACCCCTTATTTCCAAC	This work
*nadA*-R	ACGACGGCCAGTGCC AAGCTT TCACCTTCCAAGTATTAACATATTT	This work
*nadB*-F	AGGAGGTTAGTTAGA GGATCC ATGAATATTCAAACTGACGTATTAAT	This work
*nadB*-R	ACGACGGCCAGTGCC AAGCTT TCAAATGTTGACCAATTCATTTTTC	This work
*nadC*-F	AGGAGGTTAGTTAGA GGATCC ATGAATTGGTCAACATTTGATGAT	This work
*nadC*-R	ACGACGGCCAGTGCC AAGCTT TTATTTTTCATTTCTTAAGTTTTTCATGC	This work
*NadC′*-F	ACATCCCCCTTTCGC CAGCTG GAATCCATTTTGGGGGAAAAG	This work
*NadC′*-R	CAGGCTTCTTATTTTTAT GCTAGC TTATTTTTCATTTCTTAAGTTTTTCATGC	This work
pMTL-F	TGAAGTACATCACCGACGAGCAAG	This work
pMTL-R	TGCTGCAAGGCGATTAAGTTGGGT	This work
pMTL-R*′*	CCTGTTGAACCATTAGCTAAGGA	This work
Primers for RT-PCR		
*nadA*(RT)-F	CAAAGACCTGAGGTGCAGGAA	This work
*nadA*(RT)-R	GCCATTGGACAGCCAGCTT	This work
*nadB*(RT)-F	CTTCAGGCGGTATAGGTGG	This work
*nadB*(RT)-R	TCGCGAACGTCTATGTTATG	This work
*nadC*(RT)-F	AAACGCTTAGAGGGCACAGG	This work
*nadC*(RT)-R	TGCCGTCAGAAAGACCAAATC	This work
CAC2679-F	GACATTACTTCAAACGAACCTG	Liao et al. (2018)
CAC2679-R	CCCTTAGCCCATTTATTCCT	Liao et al. (2018)

### Culture conditions

*Escherichia coli* strains were cultured aerobically with Luria–Bertani (LB) medium supplemented with 20 mg/L tetracycline or/and 25 μg/mL chloramphenicol if needed, and *C. acetobutylicum* strains were cultured anaerobically with reinforced clostridial medium (RCM) (Ventura et al. 2013) supplemented with 30 mg/L thiamphenicol if needed. P2 medium (containing 80 g/L glucose) (Liao et al. 2017) was used for the batch fermentation of *C. acetobutylicum*. Both of *E. coli* and *C. acetobutylicum* were incubate at 37 °C.

### Recombinant plasmid construction and transformation

The genome DNA of *C. acetobutylicum* ATCC 824 was extracted using AxyPrep™ Genomic DNA Kit (Corning, Wujiang, China). The NADH de novo synthesis genes *nadA*, *andB*, *nadC* or *nadC’* was isolated from the genome DNA of *C. acetobutylicum* ATCC 824 by PCR, the primers used for gene amplification are shown in Table 2. Subsequently, the PCR products were purified and *nadA*, *andB* or *nadC* was inserted into the shuttle vector pMTL-Pthl, which was digested with restriction enzymes *Bam*HI and *Hin*dIII (Thermo Scientific, Shanghai, China), and *nadC′* was inserted into the recombinant plasmid pMTL-Pthl thiCGE, which was digested with restriction enzymes *Pvu*II and *Nhe*I (Thermo Scientific, Shanghai, China), then the recombinant plasmids pMTL-Pthl nadA, pMTL-Pthl nadB, pMTL-Pthl nadC and pMTL-Pthl thiCGE–nadC were obtained. The method of ligation was according to the instruction of ClonExpress II One Step Cloning Kit (Vazyme, Nanjing, China). Before transferring into *C. acetobutylicum* ATCC 824, the recombinant plasmids must be methylated by transforming into *E. coli* TOP10 (containing plasmid pAN2). Then, the methylated plasmids could be transferred into *C. acetobutylicum* ATCC 824, the transformation method was described previously (Liao et al. 2017). The transformants of 824(nadA), 824(nadB) and 824(nadC) were identified by PCR with primers pMTL-F/pMTL-R and transformants of 824(thiCGE–nadC) was identified with primers pMTL-F/pMTL-R′ (Table 2).

### RNA extraction and RT-PCR analysis

*Clostridium acetobutylicum* ATCC 824 was inoculated in liquid RCM for 12–24 h, then transferred to fresh RCM (inoculum size was 10% v/v) and cultured to logarithmic phase. The seed was inoculated in P2 medium without or with 2.0 g/L aspartate. The samples were taken at 12, 24, and 36 h and used for total RNA extraction. The method of total RNA extraction was according to the instruction of RNAprep pure Cell/Bacteria Kit (Tiangen Biotech, Beijing, China). cDNA was synthesized using total RNA as the template, and the detailed operation procedures was according to the instruction of PrimeScript™ RT reagent Kit with gDNA Eraser (Takara, Dalian, China). Real time PCR (RT-PCR) analysis used cDNA (diluted 10 times) as the template, and detailed operation procedures was according to the instruction of SYBR Premix Ex Taq II (2×) (Tli RNaseH Plus), Bulk (Takara, Dalian, China). The primers for target genes and housekeeping gene (CAC2679) (Tomas et al. 2003; Tseng et al. 2001) are listed in Table 2.

### Effect of exogenous aspartate addition on intracellular NAD(H) level of *C. acetobutylicum* ATCC 824

*Clostridium acetobutylicum* ATCC 824 was inoculated in liquid RCM for 12–24 h, then transferred to fresh RCM (inoculum size was 10% v/v) and cultured to the logarithmic phase. The seed was inoculated in P2 medium without or with 2.0 g/L aspartate. The samples were taken at 24 h and used for extraction of NAD^+^ and NADH. The bacteria were collected by centrifugation (4 °C, 12,000 rpm for 5 min) from 1 mL of the samples, then resuspend the bacteria with 0.3 mL of 0.2 M NaOH (for NADH extraction) or HCl (for NAD^+^ extraction). Subsequently, put the bacteria solution into 50 °C water bath for 10 min and transferred immediately to the ice for 5–10 min. Then the bacteria solution was neutralized with 0.3 mL of 0.1 M HCl (for NADH extraction) or NaOH (for NAD^+^ extraction). After that, the neutralize solution was centrifuged at 12,000 rpm for 5 min, and the supernatants were used for the determination of NAD^+^ and NADH (San et al. 2002).

### Batch fermentation

*Clostridium acetobutylicum* was inoculated in liquid RCM for 12–24 h, then transferred to fresh RCM (inoculum size was 5% v/v). The strain was used as seed when it grown to the logarithmic phase. To study the effect of aspartate on ABE fermentation of *C. acetobutylicum* ATCC 824, different concentrations (0, 1.0, 1.5, 2.0 and 2.5 g/L) of aspartate was added in P2 medium. For fermentation performance tests of the engineered strains, the seed was inoculated in P2 medium. The samples were taken regularly and used for determination of the cell density and the concentration of glucose and products.

### Analytical methods

Cell density was measured by UV spectrophotometer (UV2100, Unico, USA). RT-PCR was performed using fluorescent quantitative PCR (Roche, Switzerland). The sugar concentration was measured by high performance liquid chromatography (HPLC; Waters 2695, Milford, MA), and the products concentration was measured by gas chromatograph (GC; Agilent 7890A, Agilent Technologies), according to our previous study (Liao et al. 2017).

## Results

### Effect of aspartate on ABE fermentation of *C. acetobutylicum* ATCC 824

**Fig. 2:**
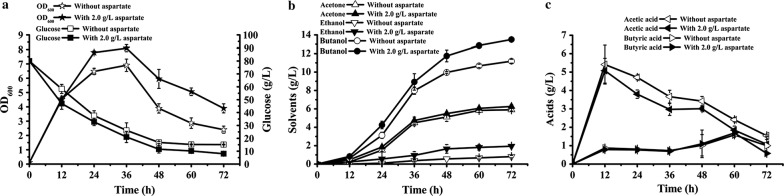
ABE fermentation with or without aspartate (2.0 g/L). **a** Growth and glucose consumption; **b** solvents production; **c** organic acids production

In order to study the effect of aspartate on ABE fermentation, batch fermentation was carried out by exogenously adding different concentrations of aspartate in P2 medium. As shown in Table 3, the max OD_600_, sugar consumption and butanol production first increased then decreased slightly with increasing the concentration of aspartate, and the best fermentation performance was obtained when the concentration of aspartate reached 2.0 g/L. Compared with the control, the max OD_600_, sugar consumption and butanol production were increased by 17.7 ± 0.4%, 12.5 ± 1.5% and 20.7 ± 2.0%, respectively. In addition, the organic acids concentration was significantly decreased by 42–43.9% (Table 3). It should be noted that the ethanol production was ~ 12 h earlier than that of the control (without aspartate) (Fig. 2).

**Table 3:** Effect of aspartate on ABE fermentation performance of *C. acetobutylicum* ATCC 824

Aspartate (g/L)	Acetone (g/L)	Ethanol (g/L)	Butanol (g/L)	Acetic acid (g/L)	Butyric acid (g/L)	Butanol yield (g/g)	Max OD_600_	Glucose consumption (g/L)
0	5.90 ± 0.14	0.80 ± 0.13	11.18 ± 0.21	1.54 ± 0.04	1.0 ± 0.34	0.172 ± 0.002	6.9 ± 0.43	64.86 ± 0.26
1.0	5.61 ± 0.55	1.85 ± 0.10	12.55 ± 0.21	1.31 ± 0.04	1.21 ± 0.24	0.180 ± 0.004	7.3 ± 0.32	69.75 ± 0.49
1.5	6.18 ± 0.25	2.06 ± 0.33	13.24 ± 0.15	1.21 ± 0.13	0.51 ± 0.05	0.187 ± 0.004	7.7 ± 0.21	70.79 ± 0.52
2.0	6.26 ± 0.20	1.94 ± 0.12	13.50 ± 0.04	1.07 ± 0.01	0.58 ± 0.01	0.185 ± 0.004	8.1 ± 0.24	72.95 ± 1.27
2.5	6.08 ± 0.17	1.53 ± 0.08	13.28 ± 0.10	1.40 ± 0.06	0.91 ± 0.06	0.188 ± 0.002	7.9 ± 0.14	70.54 ± 0.33

±, average of three replicates

### Effect of aspartate on the transcription of NADH de novo synthesis genes and the intracellular NAD(H) biosynthesis of *C. acetobutylicum* ATCC 824

**Fig. 3:**
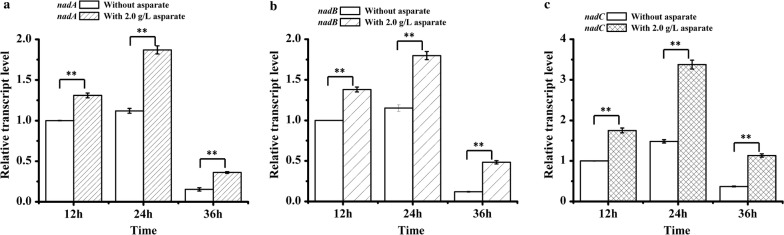
RT-PCR analysis the transcriptional level of NADH de novo synthesis genes. **a** The transcriptional level of *nadA*; **b** the transcriptional level of *nadB*; **c** the transcriptional level of *nadC*. The data are the means and standard deviations of three replicates (**P ≤ 0.01; *t* test)

**Fig. 4:**
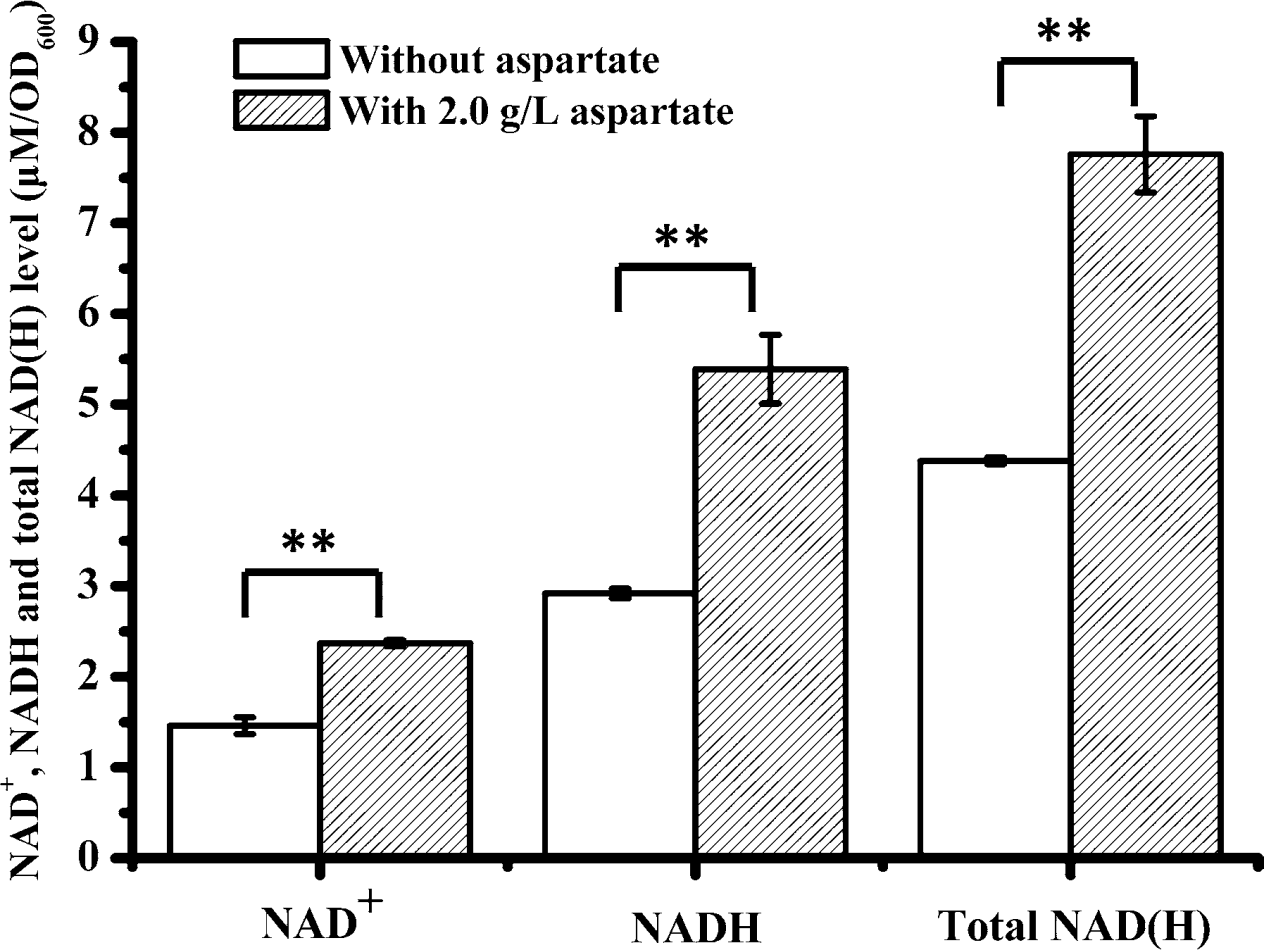
NAD^+^, NADH and total NAD(H) concentration of *C. acetobutylicum* ATCC 824 with or without aspartate (2.0 g/L). The data are the means and standard deviations of three replicates (**P ≤ 0.01; t-test)

Based on the genomic sequence analysis, we speculated that the de novo synthesis of NADH in *C. acetobutylicum* ATCC 824 is through Asp pathway, which using aspartate as the precursor. To investigate the effect of aspartate on NAD(H) synthesis, the transcription of NADH de novo synthesis genes (*nadA*, *nadB* and *nadC*) and the concentration of intracellular NAD(H) were detected when 2.0 g/L aspartate was added (no aspartate addition was used as control). As shown in Fig. 3, in general, the transcription level of *nadA*, *nadB* and *nadC* increased first and then decreased from 12 to 36 h. When 2.0 g/L aspartate was added, the transcription levels of all these three genes were consistently higher than that of the control. Furthermore, the concentration of intracellular NAD^+^, NADH and total NAD(H) were increased from 1.46 ± 0.09, 2.92 ± 0.05 and 4.38 ± 0.04 μM/OD_600_ to 2.37 ± 0.04, 5.39 ± 0.04 and 7.76 ± 0.42 μM/OD_600_, respectively (Fig. 4).

### Effect of overexpressing the NADH de novo synthesis genes on ABE fermentation

As exogenously addition of the NADH de novo synthesis precursor (aspartate) could improve the butanol production by promoting the biosynthesis of NAD(H), then we speculated that the butanol production could be promoted by enhancing the de novo synthesis pathway of NADH. Therefore, the essential genes for the de novo synthesis of NADH (*nadA*, *nadB* and/or *nadC*) has been overexpressed in *C. acetobutylicum* ATCC 824, and only three engineered strains 824(nadA), 824(nadB) and 824(nadC) were finally obtained. As shown in Table 4, the butanol titer of engineered strains 824(nadA), 824(nadB) and 824(nadC) was 11.42 ± 0.13 g/L, 11.39 ± 0.21 g/L and 11.82 ± 0.11 g/L, respectively, which were higher than that of the wild type strain (10.41 ± 0.06 g/L).

**Table 4:** Comparison of fermentation performance of *C. acetobutylicum* ATCC 824 and recombinant strains

Strains	Acetone (g/L)	Ethanol (g/L)	Butanol (g/L)	Acetic acid (g/L)	Butyric acid (g/L)	Butanol yield (g/g)	Glucose consumption (g/L)
ATCC 824	5.78 ± 0.19	0.44 ± 0.07	10.41 ± 0.06	3.83 ± 0.13	1.34 ± 0.05	0.167 ± 0.002	62.34 ± 1.21
824(nadA)	6.21 ± 0.14	1.05 ± 0.14	11.42 ± 0.13	3.23 ± 0.14	1.16 ± 0.21	0.170 ± 0.001	67.18 ± 0.58
824(nadB)	6.11 ± 0.09	0.76 ± 0.11	11.39 ± 0.21	3.36 ± 0.12	1.25 ± 0.15	0.171 ± 0.006	66.61 ± 1.04
824(nadC)	6.15 ± 0.05	0.85 ± 0.05	11.82 ± 0.11	3.05 ± 0.21	1.11 ± 0.12	0.176 ± 0.001	67.16 ± 0.64
824(thiCGE)	6.71 ± 0.16	1.85 ± 0.07	13.02 ± 0.15	1.80 ± 0.26	0.73 ± 0.11	0.186 ± 0.001	69.88 ± 0.55
824(thiCGE–nadC)	6.97 ± 0.75	1.35 ± 0.01	13.96 ± 0.11	2.46 ± 0.08	0.94 ± 0.19	0.188 ± 0.001	74.23 ± 0.52

±, average of three replicates

### Effect of enhancing the VB1 biosynthesis pathway and NADH de novo synthesis pathway simultaneously on ABE fermentation

To further improve the butanol production, VB1 biosynthesis related genes *thiC*, *thiG* and *thiE* and NADH de novo synthesis gene *nadC* were co-expressed in *C. acetobutylicum* ATCC 824. The results showed that the butanol titer of engineered strain 824(thiCGE–nadC) reached 13.96 ± 0.11 g/L, which was significantly higher than that of engineered strains 824(thiCGE) (13.02 ± 0.15 g/L) and 824(nadC) (11.82 ± 0.11 g/L), as well as the wild type strain (10.41 ± 0.06 g/L) (Table 4).

## Discussions

The co-factor NADH plays an important role in butanol biosynthesis, and insufficient intracellular NADH supply of solventogenic *Clostridium* limits the production of butanol (Li et al. 2015; Qi et al. 2018). Therefore, increasing the supply of NADH is of great significance for butanol production. Although a lot of work has been done to improve the butanol production by increasing the NADH level of solventogenic *Clostridium* in recent years (Cooksley et al. 2012; Liu et al. 2016; Qi et al. 2018; Ventura et al. 2013), there were few researches focus on increasing the NADH levels by enhancing the de novo synthesis pathways of NADH. Previous research showed that the NAD(P)H level of *Clostridium* species strain BOH3 was increased by 67% with exogenously tryptophan addition (the precursor of NAD(P)H de novo synthesis) (Li et al. 2015). When cassava hydrolysate was used as substrate for ABE fermentation, butanol titer was 68% higher than that of no tryptophan addition. This means that it could be an effective way to increase the NADH level by enhancing the de novo synthesis pathways of NADH. For de novo synthesis pathways of NADH, aspartate is the precursor of Asp pathway, while tryptophan is the precursor of kynurenine pathway (Akira et al. 2006) (Fig. 1). Based on the genome sequencing data of *C. acetobutylicum* ATCC 824 (Nölling et al. 2001), we found that it could use aspartate as the precursor for NADH de novo synthesis, rather than tryptophan. Therefore, the effect of aspartate on ABE fermentation of *C. acetobutylicum* ATCC 824 has been studied. As expected, with the increasing of aspartate concentration, the fermentation performance of *C. acetobutylicum* ATCC 824 was significantly enhanced. It was showed the optimum concentration of aspartate was 2.0 g/L, and the max OD_600_, sugar consumption and butanol production reached 8.1 ± 0.24, 71.95 ± 1.27 g/L and 13.50 ± 0.04 g/L, improved by 17.7 ± 0.4%, 12.5 ± 1.5% and 20.7 ± 2.0% compared with those of the control, respectively (Fig. 2 and Table 3). These results indicated that aspartate could promote ABE fermentation of *C. acetobutylicum* ATCC 824. The effect of tryptophan on ABE fermentation has also been studied, however, the results showed that tryptophan had an inhibitory effect on ABE fermentation (Additional file 1: Table S1).

Although we speculated that aspartate is the precursor of NADH de novo synthesis in *C. acetobutylicum* ATCC 824, it has never been proved. In order to investigate the relationship between aspartate and NAD(H), the effect of aspartate on NAD(H) biosynthesis has been studied. Firstly, the expression of NADH de novo synthesis genes has been analyzed by RT-PCR. As a result, when 2.0 g/L aspartate was added, the transcription level of *nadA*, *nadB* and *nadC* in *C. acetobutylicum* ATCC 824 were significantly higher than that of without aspartate addition (Fig. 3). In addition, the NAD(H) concentration has also been detected. The result showed that intracellular NAD^+^, NADH and total NAD(H) of *C. acetobutylicum* ATCC 824 were significantly increased when 2.0 g/L aspartate was added, which were 63.9 ± 8.0%, 85.0 ± 16.5% and 77.7 ± 8.0% higher than those of the control (no aspartate addition), and the NADH/NAD^+^ ratio was improved by 12.7 ± 2.9% (Fig. 4). These results indicated that aspartate could promote the biosynthesis of NAD(H). Therefore, the increasement of NADH concentration resulted in a reduced organic acids production, increased butanol and ethanol production, and an early production of ethanol (Table 3 and Fig. 2). For example, compared to the control, when 2.0 g/L aspartate was added, the butanol/acetone ratio was increased from 1.89 ± 0.01 to 2.11 ± 0.01, and the butanol/organic acids ratio was increased from 4.47 ± 0.62 to 8.18 ± 0.16. These results suggested that the carbon metabolism was directed to the reduced product (butanol and ethanol) synthesis under sufficient NADH supply, which were consistent with previous studies (Hönicke et al. 2012; Li et al. 2015). When 1 mM MV (an artificial electron carrier which can inhibit the hydrogenase activity) was added in the medium to increase the availability of the intracellular NAD(P)H of *C. acetobutylicum* ATCC 824, the butanol titer was improved by 23%, ethanol titer was improved by 40%, resulting in a higher butanol/acetone ratio (12.4 vs. 2.3) as compared to the control (Hönicke et al. 2012). Similarly, when 5 mM tryptophan (the precursor of NAD(P)H de novo synthesis) was added to improve the NAD(P)H levels of *Clostridium* sp. strain BOH3, the butanol titer was significantly improved and organic acids (acetic acid and butyric acid) titers were significantly reduced, which resulted in higher butanol/acetone ratio (6.6 vs. 3.7) and butanol/bioacid ratio (2.1 vs. 1.1) as compared to the control (Li et al. 2015). All of these researches indicated that the increasement of NADH had an remarkable effect on the metabolic distribution, and could promote the biosynthesis of butanol or other reducing products (BerríOs-Rivera et al. 2002; Jing et al. 2015; Li et al. 2015; Qi et al. 2018; Saini et al. 2016).

Previous studies have shown that *nadA*, *nadB* and *nadC* were the essential structural genes of the NADH de novo synthesis pathway (Haruhiko et al. 2010; Sun and Setlow 1993; Zhou et al. 2011). In addition, the expression of *nadA*, *nadB* and *nadC* were significantly up-regulated in *C. acetobutylicum* ATCC 824 after aspartate addition (Fig. 3). Therefore, the effect of overexpression of *nadA*, *nadB* and *nadC* on ABE fermentation has been studied in this study. As expected, the butanol production was improved by overexpressing these genes (Table 4). And the engineered strain 824(nadC) showed the best fermentation performance. For 824(nadC), the butanol titer was 13.5 ± 0.4% higher than that of the wild type strain, and the organic acids titer was decreased obviously (Table 4). These results indicated that with the enhancement of NADH de novo synthesis pathway, the carbon metabolism was directed to butanol synthesis. It is noteworthy that the fermentation performance of the recombinant strains overexpressing the essential genes of the de novo synthesis of NADH was inferior to that of exogenously aspartate addition, which can be attributed to the following reasons: on the one hand, single gene overexpression could not effectively strengthen the NADH de novo synthesis pathway (we have tried to co-express all of the essential genes of NADH de novo synthesis pathway, however, the recombinant vector was not successfully constructed despite a great deal of effort); on the other hand, the effect of aspartate on ABE fermentation may be attributed not only to the increasement of intracellular NADH level, but also to other factors, such as aspartate could be used as carbon and nitrogen nutrient sources or could be converted into some intermediates which were essential to cell physiology and biochemistry (Fernández and Zúñiga 2006). The beneficial effect of aspartate on ABE fermentation by *C. acetobutylicum* ATCC 824 need to be further explored.

Previous studies showed that butanol production could be significantly improved by increasing intracellular ATP and NADH levels simultaneously (Liu et al. 2016; Ventura et al. 2013). For example, the intracellular ATP and NADH levels were simultaneously increased by overexpressing of pyruvate kinase (*pykA*) gene and 6-phosphofructose kinase gene (*pfkA*), and this eventually led to a 29.4% increase in butanol production of *C. acetobutylicum* ATCC 824 (Ventura et al. 2013). The ATP and NADH levels of *C. beijerinckii* were also increased by knocking out the gene Cbei_4110, encoding the NADH-quinone oxidoreductase, which is a membrane-bound enzyme and associated with energy metabolism and electron transport, and resulted in a 21.8% increase in butanol titer (Liu et al. 2016). Our previous study showed that the sugar consumption and ATP production of *C. acetobutylicum* ATCC 824 were improved by overexpressing the VB1 biosynthesis related genes *thiC*, *thiG* and *thiE* (Liao et al. 2018), as a result, the butanol production was significantly increased. Therefore, to further improve the butanol production, VB1 biosynthesis related genes *thiC*, *thiG* and *thiE* and NADH de novo synthesis gene *nadC* were co-expressed in *C. acetobutylicum* ATCC 824 to promote the biosynthesis of NADH and ATP simultaneously. The results showed that the butanol titer of 824(thiCGE–nadC) reached 13.96 ± 0.11 g/L, which were 7.2 ± 0.4%, 18.1 ± 0.1% and 34.1 ± 0.1% higher than that of 824(thiCGE), 824(nadC) and the wild type strain, respectively (Table 4). This result indicated that the butanol production could be further improved by simultaneously increasing the levels of ATP and NADH.

In conclusion, we found that aspartate could be used as the precursor of NADH de novo synthesis to promote the NAD(H) biosynthesis in *C. acetobutylicum* ATCC 824, then resulted in an improvement in butanol production. Furthermore, the butanol production could be improved by overexpressing the NADH de novo synthesis genes. And the butanol titer could be further increased by strengthening the VB1 biosynthesis and NADH de novo synthesis pathway simultaneously. This study has a reference value for the NADH related researches of other microbes, and the engineering strategy used in this study provides a new idea for construction of efficient fuel-producing strains.

## Supplementary information

**Additional file 1: Table S1.** Effect of tryptophan on ABE fermentation performance of *C. acetobutylicum* ATCC 824.

## Data Availability

The datasets supporting the conclusions of this article are included within the article.

## Abbreviations

ABE: acetone–butanol–ethanol
LB: Luria–Bertani
RCM: reinforced clostridial medium
HPLC: high performance liquid chromatography
GC: gas chromatograph
