# Supplementary material for: The significance of aspartate on NAD(H) biosynthesis and ABE fermentation in Clostridium acetobutylicum ATCC 824
Source: AMB Express. 2019 Sep 10;9:142. doi: 10.1186/s13568-019-0874-6 (PMC6737123; doi:10.1186/s13568-019-0874-6)
Supplement: Supplementary file 1 — Additional file 1: Table S1. Effect of tryptophan on ABE fermentation performance of C. acetobutylicum ATCC 824. [file 13568_2019_874_MOESM1_ESM.docx]

AMB Express

**The significance of aspartate on NAD(H) biosynthesis and ABE fermentation in *Clostridium acetobutylicum* ATCC 824**

Zhengping Liao^1^, Xitong Yang^1^, Hongxin Fu^1,*^, Jufang Wang^1,2,*^

^1^ School of Biology and Biological Engineering, South China University of Technology, Guangzhou 510006, China.

^2^ State Key Laboratory of Pulp and Paper Engineering, South China University of Technology, Guangzhou, 510640, China.

***** Corresponding author:

Hongxin Fu (E-mail: hongxinfu@scut.edu.cn)

Jufang Wang (E-mail: jufwang@scut.edu.cn; Tel/Fax: +86-20-3938062)

**Table S1** Effect of tryptophan on ABE fermentation performance of *C. acetobutylicum* ATCC 824.

| Tryptophan  (g/L) | Acetone  (g/L) | Ethanol  (g/L) | Butanol  (g/L) | Acetic acid  (g/L) | Butyric acid  (g/L) |
| --- | --- | --- | --- | --- | --- |
| 0 | 5.90 ± 0.14 | 0.80 ± 0.13 | 11.18 ± 0.21 | 1.54 ± 0.04 | 1.0 ± 0.34 |
| 1.0 | 5.53 ± 0.06 | 2.86 ± 0.11 | 10.52 ± 0.06 | 1.67 ± 0.08 | 0.84 ± 0.11 |
| 1.5 | 5.37 ± 0.18 | 2.42 ± 0.08 | 10.26 ± 0.13 | 1.93 ± 0.20 | 1.03 ± 0.13 |
| 2.0 | 5.02 ± 0.04 | 2.11 ± 0.04 | 10.04 ± 0.17 | 1.98 ± 0.13 | 1.27 ± 0.06 |
